# A budget impact analysis of bezlotoxumab versus standard of care antibiotics only in patients at high risk of CDI recurrence from a hospital management perspective in Germany

**DOI:** 10.1186/s12913-021-06970-8

**Published:** 2021-09-09

**Authors:** Florian Jakobs, Sebastian Marcel Wingen-Heimann, Julia Jeck, Anna Kron, Oliver Andreas Cornely, Florian Kron

**Affiliations:** 1 Faculty of Medicine, Department I of Internal Medicine, University of Cologne, University Hospital Cologne, Cologne, Germany; 2 Network Genomic Medicine, University Hospital of Cologne, Cologne, Germany; 3 VITIS Healthcare Group, Cologne, Germany; 4 Faculty of Medicine, University of Cologne, University Hospital Cologne, Excellence Center for Medical Mycology (ECMM), Cologne, Germany; 5 FOM University of Applied Sciences, Essen, Germany; 6 Faculty of Medicine, Center for Integrated Oncology (CIO ABCD), University of Cologne, University Hospital Cologne, Cologne, Germany; 7 Faculty of Medicine, University of Cologne, University Hospital Cologne, Clinical Trials Centre Cologne (ZKS Köln), Cologne, Germany; 8 Faculty of Medicine, Chair Translational Research, Cologne Excellence Cluster on Cellular Stress Responses in Aging-Associated Diseases (CECAD), University of Cologne, University Hospital Cologne, Cologne, Germany

**Keywords:** Recurrent CDI, Risk factors, Bezlotoxumab, Budget impact analysis, Diagnosis Related Groups, Germany

## Abstract

**Background:**

*Clostridioides difficile* infection (CDI) is one of the leading nosocomial infections, resulting in increased hospital length of stay and additional treatment costs. Bezlotoxumab, the first monoclonal antibody against CDI, has an 1 A guideline recommendation for prevention of CDI, after randomized clinical trials demonstrated its superior efficacy vs. placebo.

**Methods:**

The budget impact analysis at hand is focused on patients at high risk of CDI recurrence. Treatment with standard of care (SoC) + bezlotoxumab was compared with current SoC alone in the 10 most associated Diagnosis Related Groups to identify, analyze, and evaluate potential cost savings per case from the German hospital management perspective. Based on variation in days to rehospitalization, three different case consolidation scenarios were assessed: no case consolidation, case consolidation for the SoC + bezlotoxumab treatment arm only, and case consolidation for both treatment arms.

**Results:**

On average, the budget impact amounted to € 508.56 [range: € 424.85 - € 642.19] for no case consolidation, € 470.50 [range: € 378.75 - € 601.77] for case consolidation in the SoC + bezlotoxumab treatment arm, and € 618.00 [range: € 557.40 - € 758.41] for case consolidation in both treatment arms.

**Conclusions:**

The study demonstrated administration of SoC + bezlotoxumab in patients at high risk of CDI recurrence is cost-saving from a hospital management perspective. Reduced length of stay in bezlotoxumab treated patients creates free spatial and personnel capacities for the treating hospital. Yet, a requirement for hospitals to administer bezlotoxumab is the previously made request for additional fees and a successful price negotiation.

## Introduction

*Clostridioides difficile* infection (CDI) is one of the leading nosocomial infections, resulting in increased hospital length of stay (LOS) and additional treatment costs. Recently published studies demonstrated an economic burden for healthcare systems of up to €50,000, especially for patients with recurrent CDI (rCDI) and patients treated in tertiary care hospitals [1]. Treatment with broad spectrum antibiotics and immunosuppressives, and having cancer as an underlying diseases are well-known risk factors for CDI [2–4].

Current international guidelines recommend the standard of care (SoC) antibiotics, metronidazole and vancomycin for mild to moderate disease stages and fidaxomicin for severe disease stages and/or multiple CDI episodes [5, 6]. Bezlotoxumab, the first monoclonal antibody against CDI, has a 1 A guideline recommendation for prevention of CDI, after the two randomized clinical trials MODIFY I/II (ClinicalTrials.gov numbers, NCT01241552, 12/11/2010 and NCT01513239, 16/01/2012) demonstrated superior efficacy over placebo [7]. Wilcox et al. demonstrated that bezlotoxumab was associated with a substantially lower rates of rCDI than placebo while having a similar safety profile. Based on pooled data from these clinical trials, two post hoc analyses showed fewer CDI-associated hospital readmissions [8] and a reduction in cumulative inpatient days [9] in patients receiving bezlotoxumab.

Health economic data regarding cost-effectiveness and the impact of bezlotoxumab on healthcare expenditures are scarce. Based on the pooled modified intention-to-treat population from the MODIFY I/II clinical trials [7], Prabhu et al. demonstrated cost-effectiveness of bezlotoxumab compared with placebo among patients receiving SoC antibiotics for treatment of CDI from the third-party payer’s perspective in the United States [8]. Comparable results were reported in a health economic evaluation from Spain [10]. As recurrence of CDI incurs significant additional treatment costs [1, 11, 12], prevention of rCDI should reduce the economic burden for healthcare systems.

Although previous studies have shown the benefit of bezlotoxumab, its use may be hampered by hurdles in reimbursement processes, such as financial risks and remuneration gaps. The current study is a budget-impact analysis of bezlotoxumab from the German hospital management perspective. The aim of this study was to analyze resource offsets attributable to disease events avoided in patients receiving SoC + bezlotoxumab versus SoC alone, and to describe pathways for efficient reimbursement strategies.

## Methods

This budget-impact analysis focused on patients at high risk to develop rCDI. Treatment with SoC + bezlotoxumab was compared with current SoC to identify, analyze, and evaluate potential cost savings from the German hospital management perspective.

### Population

The target population consisted of patients in the German inpatient setting who developed an episode of CDI and exhibited at least one risk factor for rCDI according to the summary of product characteristics of bezlotoxumab published by the European Medicines Agency (EMA) [13]. Inclusion criteria contained the following risk factors: age ≥ 65 years, one or more CDI in past 6 months, immunocompromised, severe CDI (Zar score ≥ 2), infected with a hypervirulent strain (027, 078 or 244 ribotypes), or infected with 027 ribotype. Only patients with a body weight of ≤ 100 kg were included.

### Model design

**Fig. 1:**
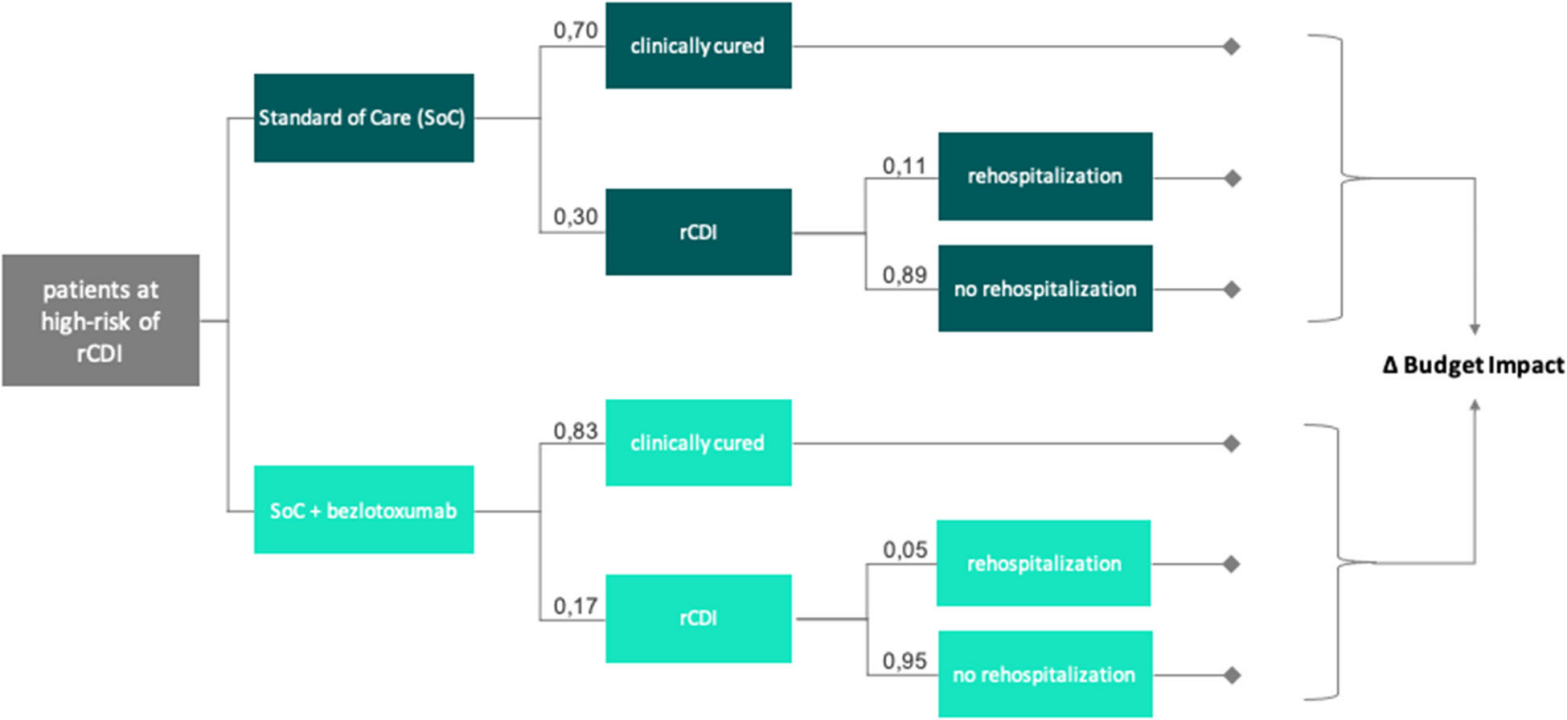
Model design including possible treatment procedures

To identify the budget impact of bezlotoxumab, the model was structured according to two different treatment arms assessing the cost savings of SoC + bezlotoxumab compared to SoC only (Fig. 1). Cost savings were weighted by the probability of occurrence of each treatment outcome. Possible treatment outcomes for patients after initial treatment were clinical cured and having rCDI. Patients were considered as clinically cured if there was no rCDI for 12 weeks after discharge. Patients with rCDI were further differentiated into those who were re-hospitalized and those who were not. Thus, the underlying time horizon was defined by the beginning of the initial treatment and the date of discharge. For patients with rCDI who have been hospitalized, the discharge date of the rehospitalization applied.

### Clinical input variables

Clinical input variables included the length of stay (LOS) of primary CDI, the rate of rCDI, the rehospitalization rate and the days to rehospitalization (counted from day of discharge of the initial hospitalization). Average LOS per German Diagnosis Related Groups (G-DRG) were considered for the underlying analysis. In case of initial treatment with bezlotoxumab, an average reduction in LOS of two days was assumed in accordance with the study of Basu et al. analyzing the hospitalization data from the MODIFY I and II clinical trials [9]. According to the EMA, a single dose of bezlotoxumab was considered by 10 mg/kg [13]. Bezlotoxumab is delivered in 1,000 mg doses and no further drug use is assumed.

For the rates of rCDI and for hospital readmission, published data was used. The rates of rCDI for SoC and SoC + bezlotoxumab, also taken from the MODIFY I and II clinical trials conducted by Wilcox et al., were 30 and 17 %, respectively [7]. Prahbu et al. further analyzed the rate of rehospitalization associated with CDI based on a pooled data set of the MODIFY clinical trials. The rate of CDI-associated hospital readmission was 11.2 % for patients receiving SoC, and 5.1 % for those receiving SoC + bezlotoxumab [14]. A specification of the G-DRG system is the so-called case consolidation (“Fallzusammenführung”) in which cases for selected DRGs are consolidated if a patient is rehospitalized within a defined period with the same diagnosis. Case consolidation leads to internal hospital costs due to rehospitalization which are not reimbursed through an additional G-DRG flat rate. In the underlying model, the variation in the days until rehospitalization led to three different scenarios: no case consolidation in both treatment arms (Scenario A), case consolidation only for SoC + bezlotoxumab (Scenario B), case consolidation in both treatment arms (Scenario C).

### Economic input variables

As the analysis was undertaken from the German hospital management perspective, only direct healthcare costs were considered. No discounting of internal hospital costs and reimbursement amounts were applied due to the underlying time period of one year, and in accordance with the German recommendation on health economic evaluation (third and updated version of the Hanover Consensus) and the General Methods (Version 5.0) of the Institute for Quality and Efficiency in Health Care (Institut für Qualität und Wirtschaftlichkeit im Gesundheitswesen, IQWiG) [15, 16]. The difference between reimbursement amount and corresponding internal hospital costs was defined herein as ‘cost savings’. All monetary information was given in Euro (€) from the reference year 2020.

Internal hospital costs and reimbursement amounts associated with SoC were determined according to G-DRG flat rates which depend and vary based on the main diagnosis, operation and procedure codes, and different LOS. The ten most common DRGs associated with the administration of bezlotoxumab were identified through the DRG browser of the Institute for the Remuneration System in Hospitals (Institut für das Entgeltsystem im Krankenhaus, InEK) [17], which comprises real-life data on DRG-level of hospitals in Germany according to § 21 Krankenhausentgeltgesetz [18]. The internal hospital costs per day were assumed to be subject to a linear cost development depending on the underlying LOS.

The appropriate value relations, which are also dependent on LOS, were taken from the DRG flat rate catalogue 2020 [19]. The corresponding reimbursement amounts resulted from the multiplication with the federal base case value of € 3,679.62 in 2020 [20].

Bezlotoxumab is currently not included in the DRG flat rate catalogue but is remunerated through an additional fee to be negotiated individually by the treating hospital. The present analysis considered € 2,950.22 for both, the purchase price [21] and the additional fee for bezlotoxumab as new treatment method (NUB, “Neue Untersuchungs- und Behandlungsmethoden”) taken from the LauerTaxe [22].

Reimbursement for rehospitalization was only taken into account if there was no case consolidation. In case of rehospitalization, costs of the same amount were assumed for both treatment arms. Both, costs and reimbursement, corresponded to the SoC arm with the same, initial LOS.

### Output

The model resulted in cost savings per treatment outcome and weighted cost savings for both treatment arms at the hospital level. Cost savings and budget impact were calculated per treated CDI patients, considering different DRGs, across the case consolidation scenarios. Due to the use of robust real-life accounting data and clinical inputs from a publicly accessible clinical trial, a sensitivity analysis was not performed.

## Results

### Cost savings per treatment outcome

Without considering the probability of occurrence of each treatment outcome, the scenario with no case consolidation (Scenario A) resulted in cost savings for all treatment outcomes across all DRGs (Table 1). The cost savings for the treatment with SoC + bezlotoxumab were higher than with SoC only. Within both treatment arms, the treatment of rCDI with subsequent rehospitalization also resulted in higher cost savings compared to clinically cured patients and those suffering rCDI without rehospitalization. The difference corresponded to the amount of cost savings of cured and non-rehospitalized rCDI patients who received SoC alone. In the event of case consolidation for SoC + bezlotoxumab (Scenario B), all cost savings remained unchanged except for patients with rCDI receiving SoC + bezlotoxumab treatment who produced additional costs of € 894.72 to € 3,732.59, depending on the DRG. In the scenario of case consolidation for both treatment arms (Scenario C), there were additional changes in cost savings with regards to patients with rCDI receiving the SoC therapy. Due to case consolidation these also led to additional costs and even exceeded the costs of SoC + bezlotoxumab.

**Table 1:** Cost savings of possible treatment outcomes per DRG and scenario [in €]

	No case consolidation (A)		Case consolidation for SoC + bezlotoxumab (B)		Case consolidation for both treatment arms (C)	
DRG	SoC	SoC + bezlo	SoC	SoC + bezlo	SoC	SoC + bezlo
B44C						
* clinically cured*^a^	720.27	1,167.96	720.27	1,167.96	720.27	1,167.96
* rCDI*	1,440.55	1,888.23	1,440.55	-2,928.39	-3,376.08	-2,928.39
G67A						
* clinically cured*^a^	313.59	933.23	313.59	933.23	313.59	933.23
* rCDI*	627.18	1,246.82	627.18	-894.72	-1,514.36	-894.72
G48A						
* clinically cured*^a^	675.04	1,334.05	675.04	1,334.05	675.04	1,334.05
* rCDI*	1,350.08	2,009.09	1,350.08	-2,652.99	-3,312.00	-2,652.99
G52Z						
* clinically cured*^a^	832.42	1,281.95	832.42	1,281.95	832.42	1,281.95
* rCDI*	1,664.83	2,114.37	1,664.83	-3,438.18	-3,887.71	-3,438.18
G77A						
*clinically cured*^a^	879.19	1,415.44	879.19	1,415.44	879.19	1,415.44
* rCDI*	1,758.38	2,294.63	1,758.38	-3,732.59	-4,268.84	-3,732.59
G77B						
* clinically cured*^a^	501.43	1,009.94	501.43	1,009.94	501.43	1,009.94
* rCDI*	1,002.86	1,511.37	1,002.86	-1,888.60	-2,397.11	-1,888.60
E42Z						
* clinically cured*^a^	825.51	1,275.08	825.51	1,275.08	825.51	1,275.08
* rCDI*	1,651.02	2,100.59	1,651.02	-3,400.44	-3,850.01	-3,400.44
E79A						
*clinically cured*^a^	537.58	1,106.68	537.58	1,106.68	537.58	1,106.68
* rCDI*	1,075.17	1,644.26	1,075.17	-1,994.88	-2,563.98	-1,994.88
T60E						
* clinically cured*^a^	417.26	980.84	417.26	980.84	417.26	980.84
* rCDI*	834.51	1,398.10	834.51	-1,442.57	-2,006.15	-1,442.57
F48Z						
* clinically cured*^a^	801.91	1,246.75	801.91	1,246.75	801.91	1,246.75
* rCDI*	1,603.82	2,048.66	1,603.82	-3,268.39	-3,713.23	-3,268.39

*Note*: ^a^From a hospital management perspective, the cost savings of clinically cured patients equals the cost savings of patients suffering from a rCDI without rehospitalization

### Cost savings weighted by the probability of occurrence

**Fig. 2:**
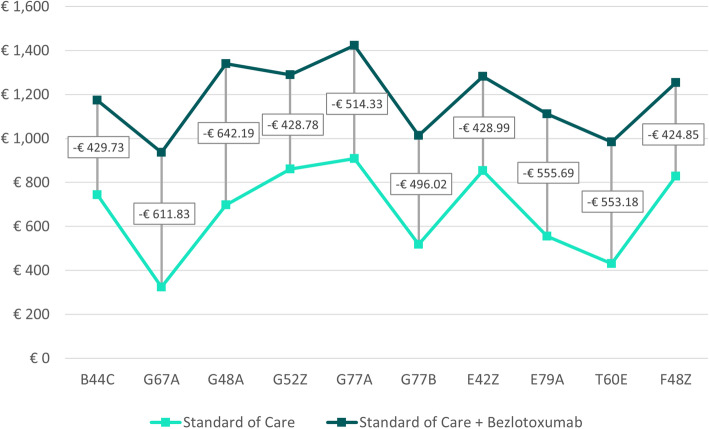
Weighted cost savings and budget impact per DRG for scenario A

**Fig. 3:**
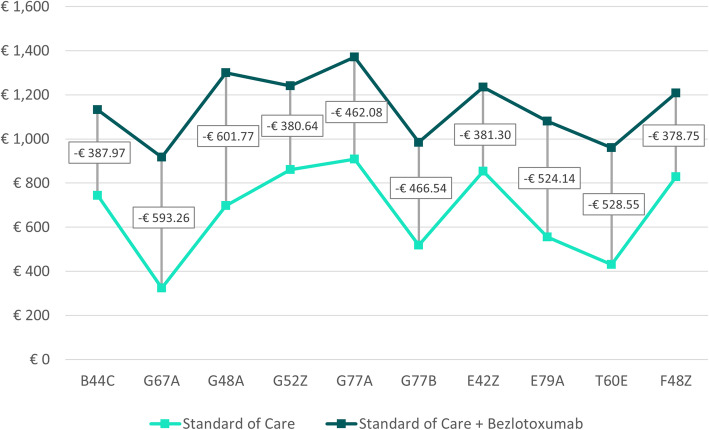
Weighted cost savings and budget impact per DRG for scenario B

**Fig. 4:**
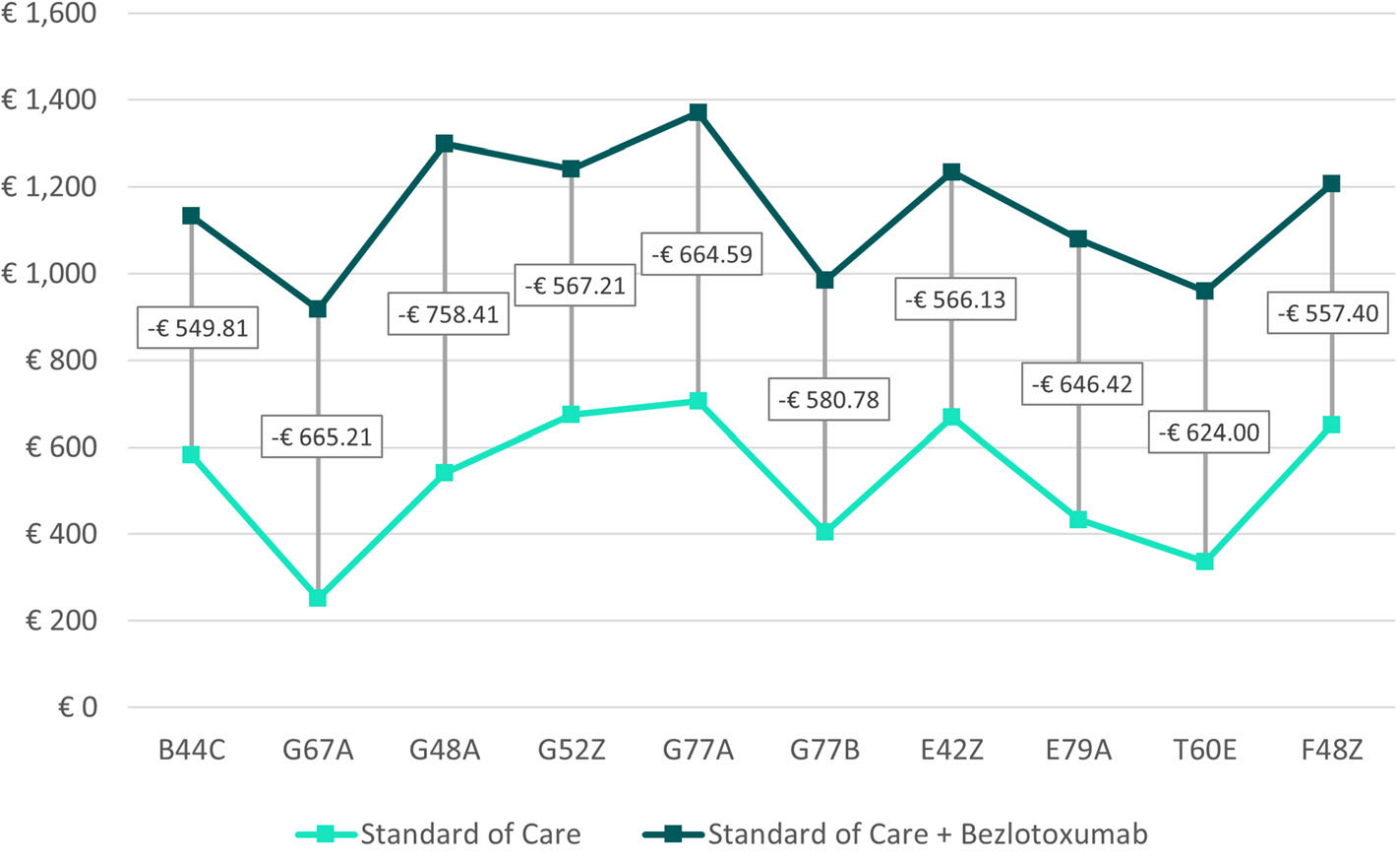
Weighted cost savings and budget impact per DRG for scenario C

Table 2 shows weighted cost savings – defined as sum of cost savings for each possible treatment outcome multiplied by the probability of occurrence (Fig. 1) - for both treatment arms across all DRGs and case consolidation scenarios. In Scenario A, the average weighted cost saving across all DRGs was € 672.27 [range: € 324.13 to € 908.73] and € 1,180.83 [range: € 935.95 to € 1,423.06] for SoC alone and SoC + bezlotoxumab, respectively (Fig. 2). Scenario B showed similar results for SoC alone and an average weighted cost saving of € 1,142.77 [range: € 917.38 to € 1,370.81] for SoC + bezlotoxumab (Fig. 3). Cost savings for Scenario C compared to Scenario B were similar for SoC + bezlotoxumab and differed for SoC therapy with an average weighted cost saving of € 524.78 [range: € 252.17 to € 706.21] (Fig. 4). DRG G77A showed the highest weighted cost saving in all scenarios, while G67A resulted in the lowest weighted cost saving.

**Table 2:** Weighted cost savings and budget impact per DRG and scenario [in €]

	No case consolidation (A)			Case consolidation for SoC + bezlotoxumab (B)			Case consolidation for both treatment arms (C)		
DRG	Weighted cost savings		Budget Impact	Weighted cost savings		Budget Impact	Weighted cost savings		Budget Impact
	SoC	SoC + bezlo		SoC	SoC + bezlo		SoC	SoC + bezlo	
B44C	744.47	1,174.21	-429.73	744.47	1,132.45	-387.97	582.64	1,132.45	-549.81
G67A	324.13	935.95	-611.83	324.13	917.38	-593.26	252.17	917.38	-665.21
G48A	697.72	1,339.91	-642.19	697.72	1,299.49	-601.77	541.07	1,299.49	-758.41
G52Z	860.39	1,289.17	-428.78	860.39	1,241.03	-380.64	673.82	1,241.03	-567.21
G77A	908.73	1,423.06	-514.33	908.73	1,370.81	-462.08	706.21	1,370.81	-664.59
G77B	518.28	1,014.29	-496.02	518.28	984.81	-466.54	404.04	984.81	-580.78
E42Z	853.25	1,282.24	-428.99	853.25	1,234.54	-381.30	668.41	1,234.54	-566.13
E79A	555.65	1,111.34	-555.69	555.65	1,079.79	-524.14	433.37	1,079.79	-646.42
T60€	431.28	984.46	-553.18	431.28	959.83	-528.55	335.83	959.83	-624.00
F48Z	828.86	1,253.70	-424.85	828.86	1,207.61	-378.75	650.20	1,207.61	-557.40
Average	672.27	1,180.83	-508.56	672.27	1,142.77	-470.50	524.78	1,142.77	-618.00

Note: Weighted cost savings = Sum of cost savings for each possible treatment outcome multiplied by the probability of occurrence (Fig. 1)

### Budget impact

The weighted cost savings shown above also resulted in consistently positive budget impact in favor of SoC + bezlotoxumab across all case consolidation scenarios (Table 2; Figs. 2, 3 and 4). On average, the impact on the budget amounted to -€ 508.56 [range: -€ 424.85 to -€ 642.19] for Scenario A (no case consolidation), to -€ 470.50 [range: -€ 378.75 to -€ 601.77] for Scenario B (case consolidation for SoC + bezlotoxumab), and to -€ 618.00 [range: -€ 557.40 to -€ 758.41] for Scenario C (case consolidation for both treatment arms). In all scenarios, F45Z was the DRG with the greatest impact on the budget, while G48A was the one with the lowest budget impact.

## Discussion

This is the first study to the best of the authors’ knowledge that assessed the budget impact of SoC + bezlotoxumab versus SoC only in patients at high risk of CDI recurrence, from a hospital management perspective in Germany. Both the medical benefit and the cost-effectiveness of bezlotoxumab have been proven in prior research [1, 7–9, 11, 12]. The study at hand, however, showed advantages from the hospital management angle and helps to put direct treatments costs into perspective. The budget impact analysis showed that the administration of SoC + bezlotoxumab compared to SoC alone led to higher cost savings across all DRGs and case consolidation scenarios. Depending on the scenario, the average budget impact ranged from -€ 470.50 to -€ 618.00 per case.

### Limitations and methodological reflection

The analysis was carried out in accordance with the highest health economic standards and using robust real-life accounting data reported to the InEK and clinical inputs from clinical trials. The latter came either from publicly available sources or high-ranked and highly recognized publications.

The analysis was carried out conservatively in order to prevent an overestimation of advantages of bezlotoxumab. For example, only a 2-day reduction in LOS was taken into account, although bezlotoxumab has been shown to lead to a reduction in LOS of 2 to 3 days, depending on the constellation of risk factors [9]. The average LOS was used as a basis for the analysis. Since the InEK uses the average LOS as reference value in the cost matrix, the resulting cost data for SoC is very robust as well. For the cost calculation of SoC + bezlotoxumab and the resulting reduction in LOS, a linear cost trend was assumed. The treatment costs are usually particularly high at the start of therapy and thus, the monetary effect of the reduction in LOS could be overestimated. A possible point of criticism is the assumed low costs of a rCDI in comparison to other studies [1, 11, 12]. The assumption of low treatment costs for rCDI patients, however, lowered the impact on the budget, since the probability of suffering rCDI is higher for patients treated with SoC compared to those receiving SoC + bezlotoxumab. The possible use of residual drugs, in this case bezlotoxumab, was also not considered. The additional fee to be negotiated for bezlotoxumab was set at the purchase price, even though additional surpluses can be generated depending on negotiating skills between hospital and health insurance companies.

### Conclusions and implications for practice

This budget impact analysis showed that SoC + bezlotoxumab compared to SoC only in patients at high risk for rCDI led to higher cost savings across all DRGs regardless of case consolidation. The cost savings were largely due to bezlotoxumab-associated reductions in LOS which in turn created free spatial and personnel capacities for the treating hospital. A requirement for hospitals to administer bezlotoxumab is a previously made NUB-request and price negotiation which has been criticized by stakeholders due to the bureaucratic effort [23]. Nevertheless, given the medical benefit and expected cost savings of up to -€ 758.41 per patient resulting from the administration of SoC + bezlotoxumab, the annual bureaucratic effort to negotiate additional fees for bezlotoxumab appears worthwhile.

## Data Availability

The datasets used and/or analysed during the current study are available from the corresponding author on reasonable request. The raw data come from publicly accessible sources.
